# Salutogenesis at Work as a Facilitator for Implementation? An Explorative Study on the Relationship of Job Demands, Job Resources and the Work-Related Sense of Coherence within a Complex Healthcare Programme

**DOI:** 10.3390/ijerph19031842

**Published:** 2022-02-06

**Authors:** Natalia Cecon, Theresia Krieger, Sandra Salm, Holger Pfaff, Antje Dresen

**Affiliations:** Institute of Medical Sociology, Health Services Research and Rehabilitation Science (IMVR), Faculty of Human Sciences and Faculty of Medicine, University of Cologne, Eupener Str. 129, D-50933 Cologne, Germany; theresia.krieger@uk-koeln.de (T.K.); sandra.salm@uk-koeln.de (S.S.); holger.pfaff@uk-koeln.de (H.P.); antje.dresen@uk-koeln.de (A.D.)

**Keywords:** work-related sense of coherence, complex interventions, implementation research, salutogenesis, job resources, job demands, qualitative data

## Abstract

Background: The implementation of complex healthcare programmes can be challenging for respective service providers (SPs) in implementation settings. A strong work-related sense of coherence (Work-SoC) promotes creation of job resources and potentially facilitates coping with demands that may arise during implementation. In this study, we analyse how SPs’ Work-SoC is influenced by job resources and demands during programme implementation and identify relevant implementation strategies to ensure a salutogenic implementation process. Methods: Qualitative data were collected during the implementation of a new complex psycho-oncological care programme called isPO. Four focus groups and four interviews were conducted with SPs. All were audiotaped, transcribed and content analysis was applied, whilst ensuring inter- and intra-rater reliability. Results: Each Work-SoC component was influenced by specific job resources and demands. In particular, comprehensibility and manageability interacted. Manageability affected assessment of the programme’s feasibility. High meaningfulness positively affected the programme’s acceptance and overall assessment among SPs. Furthermore, it buffered low manageability and was strongly associated with project identification. Conclusion: We found that Work-SoC could be used to assess SPs’ work environment, and therefore programme feasibility. It may be worthwhile to use Work-SoC as an implementation outcome or as an indicator for possible programmes.

## 1. Introduction

### 1.1. Work-Related Sense of Coherence

The Sense of Coherence (SoC) is a key concept attributed to the model of salutogenesis and stands for an orientation in life that facilitates successful coping with life events to maintain or promote health [1]. Life stressors are ultimately coped with by interactions of the SoC with one’s individual resources (general resistance resources). For the context of work, Bauer and Jenny [2] conceptualised the so-called ‘work-related sense of coherence’ (Work-SoC) as a context-specific SoC. Work-SoC is defined as the perceived comprehensibility, manageability and meaningfulness of a person’s current work situation; it is influenced by interactions of individual characteristics (i.e., personality and individual resources) with characteristics of the working environment (i.e., structures and processes) [3]. Vogt et al. [3] define the three components of Work-SoC: comprehensibility is ‘the extent to which a work situation is perceived as structured, consistent and clear’, manageability is ‘the extent to which an employee perceives that adequate resources are available to cope with the demands in the workplace’ and meaningfulness is ‘the extent to which a situation at work is seen as worthy of commitment and involvement’. Studies suggest that Work-SoC serves as an indicator of individuals’ perceptions of resourcefulness in their working situations [3,4,5]. A strong Work-SoC actively co-creates individual job resources; and inversely, with sufficient job resources, an individual is more likely to have a strong Work-SoC [4]. Hence, a person with a strong Work-SoC may be more likely to cope with job demands. However, a person with limited job resources might have a low Work-SoC, and therefore have trouble coping with challenging job demands. Further, Vogt and colleagues [3] found that coherent work experiences play a role in positive and negative health pathways. They partially mediate the relationship between job resources and job engagement, but also between job demands and exhaustion. Similarly, Vinje and Mittelmark [6] demonstrated in a study with nurses that job engagement not only leads to positive outcomes such as wellbeing or good health, but also contributes to exhaustion and burnout. Study participants tended to hold onto experiencing work-related meaningfulness then to acknowledge the importance of manageability of one’s work.

### 1.2. Implementation of Complex Healthcare Interventions

To steadily improve healthcare, it is important to successfully implement new interventions and healthcare programmes. During the implementation of healthcare interventions, especially complex programmes, challenges may arise at different levels of healthcare including end-users (e.g., patients), service providers (e.g., professional caregivers) and organisational and policy levels [7]. There is considerable risk of poor implementation if the complex intervention is not adapted to the needs of the setting and key stakeholders [8,9]. Individuals involved in the intervention may develop resistance against the programme, which in turn may lead to poor implementation [9]. Therefore, a complex intervention programme requires continuous flexible adaptation and change at both the individual and structural levels [9,10].

Service providers (SPs) are particularly involved in the implementation of new care structures and interventions. This inevitably entails both disruption of existing workflows and adaptation to new work structures. Accordingly, any innovation in healthcare can be seen as a job demand that must ultimately be coped with by the SPs in order to improve quality of care. At the same time, SPs are often confronted with health-related stressors, such as high and complex workload, shift work, being confronted with suffering and death or interprofessional communication in hierarchical structures [11]. Consequently, in alignment with literature on implementing complex interventions [9,10], we postulate that it is important for designers of new healthcare interventions and structures to consider SPs’ current job demands and resources to enable a good implementation process. Further, implementing new care structures should be accompanied by tools and actions to increase job resources, which can be physical, psychological, social or organisational resources, and thereby increase work engagement and motivation [12].

### 1.3. Objective

We suppose that the implementation of any new, complex care programme places high job demands on SPs. Therefore, for successful programme implementation, they need sufficient job resources to cope with these new demands, but also to avoid exhaustion and burnout. The Work-SoC serves as an indicator of individuals’ perceptions of resourcefulness in their working situations [3,4,5]. Low Work-SoC, resulting from high job demands and limited resources (including demands and resources before implementation and those associated with the programme itself), may not be sufficient for coping with new job demands associated with introducing and implementing a complex care programme. For example, an overly complex care programme may lead to a higher workload for SPs or misunderstanding of work processes. As a result, SPs may perceive their work as less manageable and comprehensible (Work-SoC decreases). Moreover, this may lead to a more negative assessment of the programme and dissatisfaction. Therefore, an SPs assessment of a new complex programme may depend on how successful the coping process is, which in turn is influenced by the SPs’ Work-SoC, job resources and job demands. Sufficient job resources and a higher level of Work-SoC may lead to positive coping and productive implementation. However, implementation strategies and the programme’s concept itself should hold resources for SPs to support them in the implementation process and positively affect their Work-SoC.

In this study, we aim to identify job resources and demands that affect Work-SoC during the implementation of a complex healthcare intervention. Hereby we hope to identify relevant implementation strategies that consider SPs’ resourcefulness and health at work. If programme designers, managers or funders want to facilitate the implementation process of new care structures, they might consider implementation strategies that address the context-specific job demands and resources of the care setting. Therewith, referring to use salutogenesis at work (Work-SoC and job resources) as a possible indicator for productive implementation.

## 2. Materials and Methods

### 2.1. Setting

The German healthcare system is highly complex [13,14] and often characterised as being fragmented in-patient and out-patient healthcare. The Federal Innovation Fund (IF), which was established by the Federal Ministry of Health [15], aims to improve the German healthcare system by funding innovative new forms of care that overcome these fragmentations and hence patient care.

In Germany, there are significant gaps in psycho-oncological care, which is why the National Cancer Plan calls for the integration of psycho-oncological care into biomedical cancer [16]. Unfortunately, so far guideline-based care could not be achieved on a national level in Germany [17,18,19]. In this context, the IF funded the development, implementation and evaluation of a new psycho-oncological form of care (2017–2022), called ‘isPO’ (integrated cross-sectoral psycho-oncology) [20,21]. The programme aims to (a) reduce anxiety and depression in newly diagnosed cancer patients within a 12-month period based on their individual needs and (b) implement comprehensive psycho-oncological care structures into the standard oncological care in Germany [20,21].

isPO is a new complex care programme that includes a stepped care concept, new care pathways, new psycho-oncological care networks and care process organisation, quality assurance and improvement structures, as well as a new information technology supported care documentation and assistance system called ‘CAPSYS^2020^’ which helps staff at the care networks with diverse tasks (e.g., billing, care coordination or care documentation) [20,21]. After an intensive programme development phase, in early 2019, isPO was implemented in newly established psycho-oncological care networks in North Rhine-Westphalia, Germany. Programme implementation took place at four implementation sites. These sites respectively consist of at least one certified oncological cancer centre hospital and local oncological practices which cooperate with the cancer centre hospital. These cooperations represent the psycho-oncological care networks (n = 4). Patients who receive a cancer diagnosis in the care networks are referred to the isPO programme by their treating physicians (e.g., oncologist). Based on screening instruments, patients receive psycho-oncological care that is designed to meet their individual needs [21]. Within the care networks, various professions are involved in the provision of psycho-oncological care, including licensed psychotherapists, psychosocial professionals and case managers. Additionally, isPO onco-guides, who are specifically trained cancer survivors, are also involved on a voluntary basis. Finally, care network coordinators are involved in care coordination and quality assurance. Before implementation started, all mentioned professions received training on all isPO components and their respective task areas within the programme. Afterwards, quality circles (meetings within the care networks) and quality workshops (meetings between all care networks plus programme designers) took place for quality assurance purposes and if necessary to initiate programme optimisations. Care network coordinators facilitate the quality circles where all isPO service providers may attend. Further they participate together with the clinical head of psycho-oncology as a spokesperson for their network in the quality workshops.

The isPO programme is being externally evaluated [21]. For this purpose, a study is interlinked with this new form of care to evaluate its effectiveness and quality of care. The last patient out is going to be in March 2022, therefore, final results on the main outcomes will be published elsewhere. For the presented research question, we used data collected during the programme’s first year of implementation, hence data from the formative external evaluation.

### 2.2. Design and Sample

The formative external evaluation of the new isPO programme aimed to identify both hindering and enabling factors that influence its implementation [21]. The external evaluation team was not actively involved in the implementation of the new isPO programme and a mixed methods design was used to assess patients’ and service providers’ experiences and assessment of the new isPO programme [21].

Qualitative research methods were used to gather in-depth insights into the implementation process and to understand the different professions’ opinions and experiences related to the care programme. For the presented research question of this article, we used the qualitative data set from the formative external evaluation (early implementation phase). It includes data from four semi-structured expert interviews with care network coordinators and four focus groups with isPO SPs from the respective care networks. Purposeful sampling was used [22]. Care network coordinators were interviewed individually, due to their special role in the programme (quality management, care process coordination, billing, contact person for programme designers and respective isPO service providers), which offered particularly insightful knowledge and experiences regarding the beginning of programme implementation. For the focus groups we aimed to sample diverse isPO roles and professions for each network to gain insight on all possible perspectives on the service provider level. Sometimes, due to how the programme was implemented in a particular care network, one person could represent two isPO roles (e.g., psychosocial professional and licensed psychotherapist). Table 1 gives an overview of the sample. Interviewees were either contacted directly (expert interviews) or indirectly through the care network coordinators, who forwarded our invitations to participate at the focus groups to the isPO service providers. Interviews and focus groups were conducted after roughly four months of implementation at each site (April till November 2019). In the focus groups, the number of participants varied between four and six, representing all previously mentioned isPO roles. After participants gave written consent, all interviews were audiotaped and transcribed. Expert interviews lasted between 17 and 51 min and focus groups between 80 and 90 min. The methodological procedure was approved by the Ethics Committee of the University of Cologne.

### 2.3. Interview Guidelines

The primary purpose of the interviews was to examine the implementation process, including its hindering and enabling factors. Interview questions focused on tangent job resources and demands within the implementation process. The guidelines for the expert interviews focused on the following themes: preparations for the start of the isPO programme, feasibility of the isPO programme, cooperation in the isPO programme, acceptance of the isPO programme, general evaluation of the isPO project and the potential for adoption of the isPO programme in nationwide standard care. The guidelines for the focus group were developed in accordance with the expert interview guidelines and contained similar themes; the theme of organisation of psycho-oncology before isPO was added. Table 2 depicts the guiding questions and sub-questions of the interview guidelines. Even though the guideline was not developed with the primary aim to explore job resources, demands and the Work-SoC during implementation, the guiding questions were still tangent to the topic. Data turned out very rich for the presented research question.

### 2.4. Data Analysis

Two researchers independently conducted content analysis [23] via MAXQDA 20 software (Verbi software, Berlin, Germany) [24]. One researcher was actively involved in guideline development, recruitment and data collection, whereas the other was involved in guideline development and scientific embedding. First, deductive categories were developed based on the research question. Top-level codes included work-related comprehensibility, manageability and meaningfulness; respective subcodes included resources and demands. Next, further inductive categories were coded (see Appendix A) based on the material. After each researcher independently coded the whole material, coding was discussed, and a unified categorisation system was defined based on the respective professional background of the two researchers. Accordingly, final coding was conducted by assuring inter- and intra-rater reliability, content was condensed for each code and representative quotations were selected.

## 3. Results

Below, we present our study results with a focus on top-level codes. First, we describe the job resources and demands that influenced each of the three Work-SoC components. Then we summarise the results for each component of Work-SoC and describe possible reciprocal relationships.

### 3.1. Comprehensibility

*Resources:* Information and communication management was the most-mentioned resource for work-related comprehensibility. This resource included network internal communication, as it allows for the exchange of news, demands and questions, which then in turn initiate clarification processes. Essential elements for good network–internal communication included: the establishment of regular internal isPO meetings, the organisation of informational events for peripheral professions (e.g., oncologists, nurses), implementation of a coordinating role such as the network coordinator who functions as an information ‘multiplier’ and needs-oriented communication. Most of these communication structures already existed prior to programme implementation. On the other hand, network–external contacts were found to be particularly important in supporting the implementation process. Network support could be contacted about questions and problems and forward them to responsible designer teams. SPs thus received practical information that increased both work-related comprehensibility and manageability.

*‘So it’s very good with the network support. […] it made sense […] that the networks should be supported even more by simply having two contact persons who can answer questions if there is something wrong or who can reassure people or say: Well, the topic also came up in the other network, we have found a solution, maybe this is something for you. So I find that very helpful.’*

The isPO quality workshops and quality circles were especially helpful, as they allowed exchange of information about patient care and associated challenges. Changes and solutions were initiated, many of which improved care processes. The workshops enabled SPs to receive project updates and information about important changes.

*‘That’s how it should be. That’s how the whole thing lives, through the experiences […] these circles and workshops, where you say ’OK, maybe this […] could be improved or that is difficult to handle’. If we don’t do that now, we won’t have a good programme later.*

SPs find that quality management improved comprehensibility and consequently, manageability. Moreover, information flowed faster to SPs who worked at the same location as programme designers.

The newly developed IT documentation and assistance system CAPSYS^2020^ helped to understand the programme’s complex care paths.

*‘I didn’t understand isPO until I started working with CAPSYS, I have to be honest. So all the information I got before didn’t give me the clarity that CAPSYS did. […] That was the breakthrough for me, that I knew: OK, now I know what they want from me here.’*

Lastly, the pre-implementation isPO trainings were helpful to get an understanding of the overarching concepts of the programme.

*Demands*: Information and communication management was also demanding on work-related comprehensibility, particularly in relation to information flow and lack of practical information. Information gaps reduced the manageability of SPs’ work because they negatively affected patient care coordination. Some SPs felt overloaded with information, which led to losing track of important news and changes in patient care. This reduced manageability, augmented individual programme resistance and consequently reduced work-related meaningfulness.

*‘And real life just doesn’t have that much to do with science and worlds collide there […] they are there on their science island and they have no idea what is happening here with us and what effects this flood of information, emails, calls trigger here. […] They have to think about a different communication structure […]. So, it’s no use if I make it known by email, by phone, in the workshop, in the Q-circle, everywhere, and it has zero effect. So, I really have to say that I don’t need to go to a workshop or a circle or anything. It doesn’t make sense anymore.’*

Some professions lacked knowledge about the programme (e.g., care paths, project goals) due to delayed involvement in the project. This led to low project identification during implementation and negatively affected manageability of other professions’ work.

Network–internal structures, such as personnel rotations, hindered smooth information flows and caused more work for isPO SPs. Lack of communication structures within the isPO team diminished comprehensibility and therefore, manageability.

*‘Communication is the be-all and end-all. Important points […] are discussed between doors. Our office, that’s a major problem, is a railway station. It’s a transshipment point. Everyone comes in and important things […] always happen like “Oh, by the way” […] Because it always happens so casually, one has the impression that the relevance is not that high. But it does have relevance.’*

Some SPs felt that isPO trainings were too theoretical and not practical enough. This led to ambiguities, uncertainties and dissatisfaction. Lack of practical information was, in the opinion of many SPs, due to the programme’s immaturity. Some SPs desired a contact person located in their network that would support them in the early stages of implementation.

The programme’s complexity was demanding on the SPs. They reported previous automatisms being interrupted, which causes insecurities. Some lost sight of the programme’s structure and tasks. The complexity reduced comprehensibility and consequently, possibly meaningfulness.

*‘I even lost track of how the study wants to prove the usefulness of psycho-oncology. I mean, maybe that’s not a bad thing either […] But I still wonder at which points the outcome is now assessed. I’ve lost track of that, I admit honestly, I don’t know anymore.’*

The programme’s management structure, which is supposed to help care networks in implementing and coordinating new care paths, was also reported as being too complex. It left SPs unclear on what to do within their task area.

*‘Each folder has so many subfolders, the Excel documents are very confusing from my perspective, so I lacked simplicity, practicality, where I say, okay, I have my ten-step plan here, I’ll stick to it […] this complexity should have been broken down to simple things that are easy to grasp and understand for everyone, and it was clear to me that if I gave the management structure to the staff […] I would have lost them all in the collaboration for the project.’*

The start of implementation was perceived as unstructured because pre-existing structures were not analysed by the designers and because the programme was considered immature. Therefore, SPs assessed the implementation of a few programme components as challenging. They perceived the implementation as too early, ‘unstructured’ and ‘chaotic’. This reduced comprehensibility and by extension, manageability. Some SPs wished for clearer role definitions in pre-implementation stages.

Trainings were reported as being too early in relation to implementation. SPs criticised this timing. Furthermore, new personnel did not receive extensive isPO training and depended on experienced colleagues, who are low on time resources.

Lastly, study-related changes in care paths led to changes in tasks for some SPs. This may be outside the scope of previous experiences or expertise. Therefore, low comprehensibility negatively impacted manageability.

### 3.2. Manageability

*Resources*: Individual factors, such as understanding and accepting the current situation, positively impacted work-related manageability. The SPs reported that professionality and qualifications positively influenced care quality and facilitated programme implementation. Furthermore, this led to reliability among SPs and made the programme more manageable.

SPs’ narrations indicate that strengthening human resources and organisational structures facilitated programme implementation and feasibility. Programme components were more manageable if they were similar to those of pre-existing structures. Specific factors, such as care network size, could facilitate good communication and clarification processes. Establishing new structures in terms of premises, parking permits or regular meetings also improved manageability.

*‘The two of them have really set everything up well right from the start, so that the onco-guides feel they are in good hands here, with parking cards, […] with meetings, then a group is created through which they can be called, or then a WhatsApp group is set […] we have an extra room for an onco-guide meetings. […] So everything is really very good.’*

Interdisciplinary cooperation was another important resource. When there was good information flow between oncological wards and psycho-oncology, comprehensibility and manageability increased. This in turn positively affected patient care and reduced the isPO workload for SPs. Reliable communication channels with reachable contact persons and quality workshops positively affected manageability by initiating clarification processes that led to a better understanding of one’s tasks and programme optimisation loops. Programme optimisations made it more practice-oriented and thereby increased manageability and led to improved comprehensibility of the programme, suggesting a reciprocal relationship between comprehensibility and manageability. The programme’s increasing flexibility impacted manageability by allowing for use of both initial programme care paths or flexible alternatives.

Conceptual programme-specific unique selling points also functioned (e.g., more freedom in outpatient care in isPO) as a resource.

*‘I think it’s (isPO) great. It also gives me the freedom to simply make appointments with them (patients), without having to worry about whether it’s a two-week pre-stationary, post-stationary or whatever. That gives the patients a good feeling of security, that they can contact me at any time if there is a need in between. I think that’s very good, yes.’*

*Demands*: Programme complexity negatively impacted manageability, as it hindered feasibility and reduced optimism for project success.

*‘There’s no focus on the essentials, which puts the overall goal for a really important matter at risk. […] too much is wanted too perfectly too quickly. […] So it’s like shooting cannons at sparrows, […] There is too much enthusiasm and too little pragmatism to gradually move onto the right path in small steps.’*

SPs desired reduced complexity and more practice-oriented work processes. Furthermore, the programme increased demand on resources. Lack of time and human resources, especially in networks where no new personnel were hired, was also demanding. A few SPs felt overworked. Documentation tasks and the recruitment process, in particular, led to increased workload and reduced manageability. For some SPs, lack of resources led to a perception of reduced meaningfulness and, therefore, lower programme adherence.

*’03: You can still do all that? That’s impressive. […] I can’t do that at all. […] if we had more capacity […] we could do the things you just said and at least get a few more patients, if we followed up more and approached the doctors more. But that all takes time.*

*02: We also notice that the staff situation is so thin that, […] people may tear my head off, but when a patient tells me ‘Well, the way is too long, I don’t know if I can manage to come regularly due to the distance’, then I tend to say: I understand.’*

Programme-related bureaucracy and rigid care paths overwhelmed both patients and the networks’ SPs alike.

*‘With the high level of bureaucracy, it won’t work. No hospital will be able to cope with that, to put it in a nutshell. It has to be much leaner and easier for the patient, but also for the administrative apparatus.’*

Unstructured implementation was demanding on manageability due to information deficits, which increased programme resentment. SPs felt the programme was not ready for implementation; therefore, SPs criticised the project’s time frame.

*‘It can’t be that now, […] we have the system (documentation system) in such a way that […] we can work with it, and we can discover the first teething problems. That is too late. […] getting rid of teething problems now not only costs time, it costs patients (recruitment), it costs money in the end and that is a great pity, I would say. We could have discovered many errors beforehand […] The test phase should have been a bit longer at the beginning.’*

Unreliable cooperation between different professions (e.g., fluctuating dependability of oncologists) reduced manageability.

*‘We don’t get any normal psycho-oncology consultations anymore. So that has had a real deterrent effect. […] This has somehow […] flipped a switch for them. […] and since I was in the morning department meetings and promoted the programme, we haven’t had any consultations at all since then. That has completely fallen asleep.’*

SPs reported trying to compensate for this by taking over oncologists’ work. They felt high pressure due to patient recruitment, which resulted in feelings of ‘not making it’ and affected communication with patients.

*‘I often have the impression that patients look at me questioningly, like: What does she get out of it if she signs me up now?’*

Large networks potentially have long distances over which to coordinate patient care. This is time consuming, and the patient area is large, which made it difficult to make appointments for patients who lived far away.

Another organisational demand was the lack of specific working structures. For example, clear definition and organisation of tasks was needed to avoid diffusion of responsibility.

*‘Where I always see a problem, is when we are contacted about a patient, it always goes to the general e-mail address […] and then none of the four of us feels addressed. That really annoys me. I see for myself how long e-mails remain unanswered. […] no one feels personally addressed. […] I’ve already mentioned that I don’t like it when everything is sent out via this distribution list or this e-mail address, because it simply leads to confusion. You don’t know if the things are done, who has done it now.’*

Some SPs identified strong top-down programme development as the cause of the rising demands during implementation, stating that pre-implementation analysis of the network structures and resources would have improved manageability and shortened the optimisation phase. They desired more opportunities to participate in the development phase.

*‘As peripheral locations, we were only asked when the project was ready to go. There was nothing more that could be changed, because everything had already gone through the ethics committee, so it would certainly have made sense to involve the people who work in practice in the conceptual designing process, because then I think some things would not be such huge hurdles for patients.’*

Some programme components were less manageable. For example, the care concepts of the programme focused on behavioural therapy-oriented interventions, even though SPs caring for patients have a variety of different therapy backgrounds. While programme optimisations led to a more mature programme with increased flexibility for the SPs, sometimes conflicts with designers slowed down the solution processes.

### 3.3. Meaningfulness

*Resources:* Achieving sustainable psycho-oncological care structures was perceived as a strong resource for meaningfulness. SPs desired determination of financial advantages and positive evaluation results on the programme’s effectiveness, since these would encourage health insurance providers to finance psycho-oncological care in standard care. Furthermore, they would feel their efforts were recognised.

SPs showed high project identification due to patient benefits and meaningful project goals, such as improved access to and continuity of care. SPs found the challenges in programme implementation to be manageable if patient benefits were clear and meaningful.

*‘I just remembered a picture of a really beautiful chestnut. When it’s on the tree, it has lots of thorns, but when you pick it out, you have something delicious. I think we’re still working on picking them apart and then getting at all these thorns/ well, no, and then just seeing how we manage to get at this good stuff. And it’s partly very exhausting, but I think what comes out at the end is good and […] that’s also what drives us, this commitment that we put into it, because we know, okay, we have to go through it now, but what comes out will be a good thing.’*

SPs hoped for acceleration of political processes, which have been laborious thus far, towards strengthening psycho-oncology in Germany. Because of this high project identification, the programme’s assessment was very positive in terms of meaningful project goals, but also critical due to reduced manageability during the programme’s implementation.

*‘It’s a sensational idea with a sensational goal and we were or are all very motivated to make sure that it really works and that it works somehow and that’s why I think it’s so fundamentally important and right that this is evaluated again, so that this is given a status in society, with the health insurance companies, wherever, so that it’s clear that this is simply important. Many patients need it. I would say that the concept and implementation could be improved.’*

Certain programme components (e.g., the network coordinator’s role or participatory quality management) were helpful for SPs and therefore perceived as meaningful. These allowed for better comprehensibility and manageability.

The perceived engagement of designers and cooperating health insurance companies were meaningful for SPs’ work. Oncologists’ engagement increased in the short-term with the introduction of monetary incentives per enrolled patient. In the long-term, however, oncologists’ engagement was primarily affected by their perception of meaningfulness of their role.

*‘Well, I know for sure that if the patients are well cared for psycho-oncologically […] that saves me time in the end. […] if I have a 30-min appointment, 20 min of it is counselling […] if I know that there is someone there who is the contact person and the patient is also tied up there, then I can of course say ‘Wow, that is really a heavy shoe that you are wearing. Thank God you have a contact person’. […] you get something back in return for what you invest.’*

Overall, SPs with readiness for change or pre-isPO attempts at change, as well as those who felt like they had fun at work, showed more motivation and engagement. This led to positive evaluations of their work within the programme.

*Demands:* Most demands on meaningfulness were rooted in individual attitudes and programme-specific challenges. Medical personnel reported lacking meaningfulness in their work tasks within the programme, which led to reduced manageability for isPO SPs.

*‘I would say 80 percent of the doctors smile at this project because they simply see the importance of psycho-oncology quite differently. […] If they could decide what they would do for themselves it would be not recommending patients, and that’s why I don’t believe that anything will change for the better during the project period.’*

For some SPs, dissatisfaction with programme implementation or their roles within the programme resulted in expectations of negative outcomes. However, reduced manageability and comprehensibility due to the complexity of the programme and/or conceptual aspects resulted in the reduction in meaningfulness.

Increased demands on time and human resources negatively affected meaningfulness when the perceived ratio of effort-to-benefits was unbalanced and resulted in negative programme assessment or decreased programme adherence.

*‘I have a huge time investment, the onco-guides have a huge time investment by having to deal with it all the time and I have nothing, no result. So now I have partly switched to conducting the onco-guide talks myself, which then puts even more strain on my time resources.’*

For quality management staff in particular, economic efficiency was a deciding factor for meaningfulness and programme assessment.

Furthermore, delayed implementation start dates had a negative impact on meaningfulness in terms of motivation and engagement.

### 3.4. Summary of Results

The SPs’ experiences emphasised the role of information and communication management as both a resource and a demand for work-related comprehensibility. Good internal and certain external communication paths facilitated comprehensibility. These paths allowed SPs to receive practical information and exchange information about demands and questions, which in turn initiated solution processes. This made work clearer and easier to understand, in addition to increasing manageability. On the other hand, information flow was not always needs-oriented. Information gaps reduced comprehensibility and hence, manageability. Structural demands, such as personnel rotations or lack of communication paths, hindered information flow. The programme’s complexity reduced comprehensibility and therefore, manageability and meaningfulness.

SPs described reciprocal relationships between work-related comprehensibility and manageability, which are mainly connected by good communication structures. Programme complexity, in combination with lack of resources and structures, seemed to drastically reduce work-related manageability and thus, programme feasibility, leading to negative programme assessments. As a result, programme complexity also affected work-related meaningfulness. Other professions’ lack of meaningfulness negatively affected SPs’ manageability. Low participation in the programme’s development reduced manageability of programme components in the implementation phase due to immaturities.

SPs’ high project identification due to patient benefits and project goals seemed to primarily influence their work-related meaningfulness. High meaningfulness positively affected programme assessment. At the same time, it also seemed to buffer low manageability or comprehensibility to some extent. High complexity and low manageability still negatively influenced meaningfulness, and in some cases affected programme adherence.

**Figure 1:**
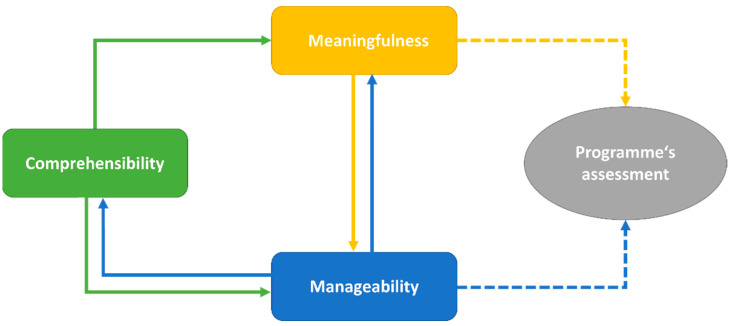
Relationships between Work-SoC’s components and SP’s assessment of the programme.

All in all, reciprocal relationships between the components are identifiable (Figure 1). We found that Work-SoC’s comprehensibility greatly influenced manageability, which in turn strongly influenced meaningfulness. Comprehensibility impacted meaningfulness somewhat directly, but mostly indirectly through manageability. A strong feeling of meaningfulness led to an increase in perceived manageability. Lastly, we found that meaningfulness and manageability influenced the SPs’ perceptions and assessments of the programme.

## 4. Discussion

Within the implementation of the new complex psycho-oncological care programme isPO, SPs were confronted with new job demands. Diverse job resources, either already existing or facilitated by the programme, helped to cope with those demands. We were able to identify what kind of job resources and demands influence SP’s Work-SoC and its components. Further, we found fluid reciprocal relationships within SPs’ Work-SoC. Work-related manageability and meaningfulness mostly influenced SP’s programme assessment, which in turn affected programme adherence. Based on the presented data we identified four superordinate themes as relevant for the implementation of complex healthcare programmes. They are discussed in the following sections and based on which exemplary recommendations for action for psycho-oncological care and a possible nationwide implementation of isPO are given whilst considering the Work-SoC.

### 4.1. Communication and Information

Our results suggest that work-related comprehensibility can be strengthened by simplifying programme complexity. This can be achieved by focusing on practical relevance in information flows and creating clear communication structures within both the implementation and design settings. We deduce that a programme’s complexity should be broken down into comprehensible target-oriented information to help the SPs to perceive their working situation as structured, consistent and clear. Moreover, effective and supportive communication management seems to be vital for SPs’ work to be comprehensible, which leads to higher manageability. Understanding and making sense of the intervention happens at both the individual and collective levels, allowing information to be integrated with experience, clinical context and pre-existing practice approaches [25]. Facilitating this process by developing and using platforms for communication, such as quality circles and quality workshops in isPO, improves confidence for interdisciplinary collaborations, team relationships, self-efficacy (individually and collectively) and shared decision-making [25]. Due to the importance of supporting communication in the implementation process [9], we recommend including an analysis of information and communication structures for possible improvements as part of a stakeholder analysis prior to programme development [26]. Furthermore, comprehensibility may be strengthened by training SPs near the start of implementation and involving all potentially affected SPs.

### 4.2. Organisational and Social Resources

Our findings show that Work-SoC’s manageability is strongly influenced by individual and organisational resources and demands, such as human resources and social capital. When implementing complex interventions, allocating enough resources (e.g., time, personnel) is important [9], as it makes the intervention more likely to be assimilated [27] and ensures fidelity [28]. Our results support these findings, suggesting that assuring enough resources increases feasibility by enabling adequate coping with any additional work resulting from the programme’s complexity. At the same time, healthcare systems face severe resource constraints, such as worsening workforce shortages [29,30], that highlight the importance of analysing and considering resources and needs prior to programme implementation. In the data we collected, climate and social aspects in the implementation settings functioned both as job resources and demands, affecting work-related manageability. This aligns with research stating that social networks and communications have a complex role in the implementation process [9,31,32]. Hence, building these social relationships, as supported by isPO’s quality assurance activities, can positively influence and facilitate implementation effectiveness [33,34].

### 4.3. Programme Development and Participatory Elements

Research on the implementation of healthcare interventions shows that ‘teething problems’ often occur, and programme optimisations are needed to improve effectiveness, acceptability and feasibility [10]. Thus, participation and engagement of stakeholders is considered helpful [35]. This was also reported by the SPs because allowing for flexibility and optimisations made work more manageable, increased feasibility and satisfaction with programme-related work. Furthermore, SPs believed participatory elements should have already been part of the programme’s development phase, as proposed by research [36]. They identified many challenges during isPO’s implementation attributable to the programme’s development phase. Participation during a programme’s development phase could therefore be helpful for detecting conceptual strengths and weaknesses early on [8], and by this facilitate work-related comprehensibility and manageability and consequently the implementation process. A comprehensive understanding of all concerned stakeholders in the respective setting is needed, as it leads to internal knowledge and understanding of implementation barriers and enablers [37,38,39]. Therefore, it is important to include enough resources in terms of time and personnel for the inclusion of participatory elements in a programme’s development [40,41].

### 4.4. Ambivalent Function of Work-Related Meaningfulness

We found that work-related meaningfulness buffered low manageability. High project identification strongly facilitated work-related meaningfulness and promoted individual engagement and motivation to overcome manageability challenges. While this may be good for a programme’s implementation, it might be a risk for SPs if job demands are straining them for too long. Especially high programme complexity in combination with low availability of personnel resources may not always be buffered by high project identification and thus, partially reduces meaningfulness and drains SPs. Jenny and colleagues [42,43] describe similar salutogenic and pathogenic pathways as partially found in our data. Within the salutogenic path, job resources can lead to growth and development and thereby to positive health (physical, mental and social self-fulfilment). Based on our data, we could identify job resources that strongly associate with self-fulfilment, e.g., engagement, motivation, project identification or fun at work. Whereas negative health is determined by job demands that may lead to loss and deterioration (impaired physical, mental and social self-reproduction). Here, we identified job demands that are mainly associated with the reduction in comprehensibility and manageability, e.g., lack of human resources or coherent work structures, but we were also able to identify negative consequences on the SPs emotional level, e.g., insecurities, helplessness or exhaustion. This aligns with existing study results suggesting that job demands deplete resources and eventually lead to burnout, whereas job resources foster engagement while buffering negative effects of job demands [44]. Therefore, it is even more important to secure enough resources for the SPs. These study results as well as the results presented in this article suggest [45] that job resources and demands need to be in an appropriate load-balance for SPs to manage work in a healthy way. Further studies suggest that meaningfulness should be given a more prominent role in this subject [6,46,47,48]. On the one hand, meaningful job experiences result in the wish to protect these experiences, which again enhances salutogenic processes [49]. On the other hand, meaningfulness in terms of job engagement may contribute to exhaustion and burnout [6]. SPs’ wishes to uphold meaningfulness can contribute to a strong sense of duty and self-demand on one’s work, thereby, distress, overload and fatigue may be intensified which can lead to burnout. A recent study on the sense of coherence and burnout in healthcare professionals during the COVID-19 pandemic supports this, showing that the dimension of meaningfulness had the highest predictive value for professional burnout [50].

In addition, we observed that high meaningfulness led to positive programme assessment, such as it being a good overall idea or an important goal. Low manageability, however, led to negative assessments of the programme’s feasibility and even partially led to reduced programme adherence. Furthermore, SPs reported on another group of SPs who showed low work-related meaningfulness in relation to isPO. This led to implementation problems for SPs with high meaningfulness and reduced their work-related manageability. Therefore, it may be worthwhile to improve social cohesion to increase meaningfulness in all involved SPs and thereby cooperation among SPs. This aligns with Luig et al. [25] (p. 441) who posit that engagement ‘allows constructive cross-fertilisation between implementation projects and contextual elements (organisational context, research and theory and policy and funding)’.

### 4.5. Practical Implications for Psycho-Oncological Care

Based on the discussed themes that are relevant for the implementation of complex healthcare programmes, Table 3 depicts possible recommendations for actions to improve job resources and the Work-SoC of service providers in current psycho-oncological care structures as well as in regard to the newly developed programme isPO and its possible implementation into nationwide care.

### 4.6. Limitations and Strengths

Although applying two different qualitative data collection methods helped generate detailed insight into the SPs’ experiences with programme implementation, and purposeful sampling led to the inclusion of all isPO care networks and roles, we may have been unable to capture the comprehensive attitudes from medical personnel, as they were underrepresented. Furthermore, the guidelines for the interviews focused primarily on retrieving data about the implementation process for the purpose of conducting an external formative evaluation. Possibly, even more detailed results could have been obtained if the interview guidelines had solely focused on Work-SoC. Nevertheless, we were still able to obtain rich qualitative material for analysis. To our knowledge, this was the first study to explore the relationship of SPs Work-SoC in the context of implementing complex care programmes and hereby contributing a salutogenic perspective to the existing literature and recommendations on the implementation of complex healthcare interventions. By applying such a perspective, stakeholders who aim to implement new structures may facilitate the implementation process, because changes in the care setting are introduced more in regard to the setting’s needs. We find that the results’ transferability to other SP groups and care settings may benefit from more comprehensive studies, in the sense of a sequential exploratory design, using quantitative methods next.

## 5. Conclusions

The implementation of complex healthcare structures is challenging for SPs, since their working structures are changing, and routines are being disrupted. SPs’ Work-SoC, which is influenced and cocreated by job demands and resources, facilitates coping with the implementation process. Simultaneously, implementation strategies can influence this coping process by adding to pre-existing job demands and resources, thus influencing SPs’ Work-SoC and in doing so, affecting their health by contributing to salutogenic or pathogenic pathways. In this study, we raise awareness of these interrelations and would like to promote utilising the Work-SoC as a tool and indicator to (i) assess SPs’ perception of work environment and thus facilitate the implementation process of new structures and (ii) explore the new intervention programme’s feasibility across different settings. Both are important for a new programme’s development, optimisation and implementation. As a programme designer, hospital manager, politician, etc., it is important to consider how to successfully improve quality of care. Therefore, considering the topics discussed in this article and hereby finding ways of improving Work-SoC we recommend careful consideration of SPs’ needs and Work-SoC. When developing and implementing new complex intervention programmes, measuring SPs’ Work-SoC before, during and after programme implementation would be beneficial, as it may facilitate exploration of optimisation needs and consequently positively impact the implementation process and quality of care.

## Figures and Tables

**Table 1:** Sampling and data collection method.

Care Network	Research Method	Number of Interviewees	Place of Data Collection	Role(s) in isPO
1	Expert interview	1	Office, at the care network	Care network coordinator
	Focus group	4	Conference room, at the care network	Psychotherapist Psychosocial professional Case management
2	Expert interview	1	Office, at the care network	Care network coordinator
	Focus group	5	Conference room, at the care network	Oncologist Psychotherapist Psychosocial professional Deputy care network coordinator Case management
3	Expert interview	1	During a quality workshop, at the medical association North Rhine	Care network coordinator
	Focus group	6	Conference room, at the care network	Oncologist Psychotherapist Documentation assistant Case management
4	Expert interview	1	During a quality workshop, at the medical association North Rhine	Care network coordinator
	Focus group	5	Conference room, at the care network	Psychotherapist Psychosocial professional Case management

**Table 2:** Interview guideline questions.

Guiding Questions	Sub-Questions
Period before start of the project	
From your point of view, how well was psycho-oncological care organised before isPO?	Were there efforts to change something before?
Implementation	
Please tell us how you experienced the preparations and start of the isPO project.	How did the trainings go? Were they sufficient or is further training necessary? Did you feel sufficiently informed (at the start of the project)? (Were there contact persons available to clarify any questions?) How did you experience the introduction of the stepped care programme? How do you assess the human resources for the project in your institution or in your area of responsibility?
How do you experience the feasibility of the isPO-programme so far?	Which aspects are easy to implement? What problems or complications have you encountered? (Can you give examples?) How do you experience the handling of CAPSYS? * How well do you think the isPO programme fits into the existing work processes and structures? How do you proceed if you have further questions about the intervention and its implementation? How confident are you that the obstacles/difficulties in the implementation can be overcome? What gives you this confidence (or lack of confidence)? Do they feel that you can actively contribute to the programme’s success? What do you think could be changed in the implementation process?
Cooperation and communication, acceptance and attitudes	
How do you experience the cooperation and communication in isPO?	How is the cooperation and communication… … between you, the isPO service providers? … between you, the isPO service providers and hospital staff (doctors, nurses etc.)? … between you, the isPO service providers and the staff of the local oncological practices (doctors, medical assistants, etc.)? … between you, the isPO service providers and project staff/“isPO developers”? How do you experience the cooperation in the quality circles? Which topics were/are present in the quality circles?
How do you experience the project’s acceptance in your care network?	How do you assess the acceptance of the referring doctors, for example?
Outlook and conclusion	
All in all, how would you asses the project?	How do you rate the time required? How do you rate the new care structure and the stepped care system?
How do you assess the potential of isPO to be adopted into nationwide routine care?	What concrete measures do you think would increase this potential? What do you think is important to bring isPO out of “project status” and into mainstream care?
We would like to take this opportunity to thank you for this focus group discussion/interview. Finally, is there anything that you have not yet mentioned that you would like to tell us? Or something you would like to comment on? CAPSYS2020 is the newly developed care documentation and assistance system for the isPO programme.	

**Table 3:** Exemplary recommendations for action for psycho-oncological care.

Recommendation	Possible Advantages and Effects
Establish sufficient, consistent and sustainable funding for psycho-oncology *	Hereby improving resources, e.g., infrastructure or personnel which may lead to better manageability of work for staff and less risk for overworking and burnout
Invest in sufficient training of psycho-oncologists (not just psychotherapists **)	Hereby making sure enough qualified personnel is available to care for patients
Conduct a stakeholder analysis before implementing new psycho-oncological structures at a new implementation setting/your care site	Current job resources and demands can be identified and thereby existing structures and setting-specific needs considered
Focus on practical relevance in information flows	Making information structured and concise in accordance with the stakeholders’ needs facilitates comprehensibility and hence feasibility
Implement a reliable contact person and/or support system for the implementation of new structures, especially at the beginning	Improves work-related comprehensibility and manageability and facilitates the implementation process
Allow for bottom-up (participatory) processes	By including stakeholders in the implementation and optimisation processes the programme is adapted to the settings needs, hereby improving Work-SoC and programme acceptance, e.g., by promoting communication and exchange in the form of participatory quality management
Augment the visibility and benefit of psycho-oncology	Peripheral stakeholders who also work in oncological care (e.g., oncologists or nurses) are hereby not only better informed, but also meaningfulness and cooperation may be improved. This may support psycho-oncological service providers in their work’s manageability and facilitate patients’ access to psycho-oncological care

* currently, in Germany psycho-oncological care is not systematically funded as other health services; ** in regular German outpatient psychotherapeutic care patients are faced long waiting times to receive care. The majority of psychotherapists do not have specific psycho-oncological qualifications, which makes access to adequate care even more challenging for patients.

## Data Availability

The data are not publicly available due to ethical and legal restrictions, as participants of this study did not agree for their data to be shared publicly.

## Appendix A

**Table A1:** All subcodes of the final coding system.

Work-related comprehensibility	Resources	Information and communication management	Network internal communication
			Contact persons
			Quality workshops
			Quality circles
			Closeness to designers
		Easy usage of CAPSYS^2020^ *	
		Trainings	
	Demands	Information and communication management	Flow of information
			Lack of practical information
			Lack of contact persons
			Network overarching exchange
		Programme and study complexity	Management structure
		Unstructured start of implementation	Lacking analysis of existing structures
			Too early implementation
			Lack of definitions of roles and tasks
			Trainings
			Incomplete induction of new staff
		Changed role	
Work-related manageability	Resources	Personnel	isPO-onco guides’ engagement
			Situation acceptance
			Professionalism/qualification
			Increase in personnel resources
			Personnel resources PSF *
		Organisational structure	Pre-existing similar structures
		Interdisciplinary cooperation	
		Communication structure	Reachable contact persons
			Quality workshops
		Network support	
		Increasing flexibility	Leaving new care paths
		More freedom in outpatient care	
		Feasibility of the PSF * role	
	Demands	Programme’s complexity	Increased demands on resources due to higher workload
			Rigidity of care paths
			Bureaucracy
		Unstructured start of implementation	Information deficits
			Immature programme
			Trainings
			Ongoing changes
		Unreliable cooperation	
		Lack of personnel resources	
		Organisational structures	Spatial distance
			Lacking/unfavourable working structures
		Documentation in CAPSYS^2020^	
		Top-down programme development	
		Care concept	
		Recruitment pressure	
		Conflicts with designers	
Work-related meaningfulness	Resources	Sustainability of psycho-oncology	Refinancing
			Effectiveness results
			Confidence for the future of psycho-oncological care
		Project identification	Patient benefits
			Project goals
		Programme’s conception	
		Engagement	Insurance companies’ engagement
			Psycho-oncological personnel’s engagement
		Advantages for physicians	Better manageability
			Monetary incentives
		Need for change	
		Fun at work	
		Constructive exchange with stakeholders	
	Demands	Individual attitudes	Medical personnel
			Negative expectations
			Deviating work attitudes
		Programme’s concept and complexity	Low manageability of programme components
		Time and personnel expenditure	
		Lack of refinancing	
		Stigma around psycho-oncology	
		Late start of implementation	

* PSF stands for psychosocial professional. They are part of the isPO service provider team; CAPSYS^2020^ stands for the newly developed documentation and assistance IT-system that was implemented within the isPO programme.

## References

[1] Antonovsky A. (1987). Unraveling the Mystery of Health: How People Manage Stress and Stay Well.

[2] Bauer G., Jenny G., McIntre S., Houdmont J. (2007). Development, implementation and dissemination of occupational health management (OHM): Putting salutogenesis into practice. Occupational Health Psychology. European Perspectives On Research, Education and Practice.

[3] Vogt K., Jenny G.J., Bauer G.F. (2013). Comprehensibility, manageability and meaningfulness at work: Construct validity of a scale measuring work-related sense of coherence. SA J. Ind. Psychol..

[4] Broetje S., Bauer G.F., Jenny G.J. (2020). The relationship between resourceful working conditions, work-related and general sense of coherence. Health Promot. Int..

[5] van der Westhuizen S.C. (2018). Incremental validity of work-related sense of coherence in predicting work wellness. SA J. Ind. Psychol..

[6] Vinje H.F., Mittelmark M.B. (2007). Job engagement’s paradoxical role in nurse burnout. Nurs. Health Sci..

[7] Ferlie E.B., Shortell S.M. (2001). Improving the quality of health care in the United Kingdom and the United States: A framework for change. Milbank Q..

[8] Levati S., Campbell P., Frost R., Dougall N., Wells M., Donaldson C., Hagen S. (2016). Optimisation of complex health interventions prior to a randomised controlled trial: A scoping review of strategies used. Pilot Feasibility Stud..

[9] Damschroder L.J., Aron D.C., Keith R.E., Kirsh S.R., Alexander J.A., Lowery J.C. (2009). Fostering implementation of health services research findings into practice: A consolidated framework for advancing implementation science. Implement. Sci..

[10] Sermeus W., Richards D.A., Rahm Hallberg I. (2015). Modelling Process and Outcomes in Complex Interventions. Complex Interventions in Health: An Overview of Methods.

[11] Eurofound (2012). Fifth European Working Conditions Survey.

[12] Bakker A.B., Demerouti E. (2007). The Job Demands-Resources model: State of the art. J. Manag. Psychol..

[13] Döring A., Paul F. (2010). The German healthcare system. EPMA J..

[14] Busse R., Blümel M. (2014). Health System Review. Health Systems in Transition. Health.

[15] Gemeinsamer Bundesausschuss Innovationsausschuss Innovationsfonds. https://innovationsfonds.g-ba.de.

[16] European Partnership for Action Against Cancer National Cancer Plan Germany. http://www.epaac.eu/from_heidi_wiki/Germany_Working_Document_on_NCP_German_4.1.2012.pdf.

[17] Bergelt C., Reese C., Koch U., Mehnert-Theuerkauf A., Koch U. (2016). Psychoonkologische Versorgung in Deutschland. Handbuch Psychoonkologie.

[18] Schulz H., Bleich C., Bokemeyer C., Koch G., Härter M. Psychoonkologische Versorgung in Deutschland: Bundesweite Bestandsaufnahme und Analyse. https://www.bundesgesundheitsministerium.de/service/publikationen/gesundheit/details.html?bmg%5Bpubid%5D=3273.

[19] Mehnert A., Koranyi S. (2018). Psychoonkologische Versorgung: Eine Herausforderung. Dtsch. Med. Wochenschr..

[20] Kusch M., Labouvie H., Schiewer V., Talalaev N., Cwik J.C., Bussmann S., Vaganian L., Gerlach A.L., Dresen A., Cecon N. (2021). Integrated, cross-sectoral psycho-onoclogy (isPO): A new form of care for newly diagnosed cancer patients in Germany. 2021, in press. BMC Health Serv. Res..

[21] Jenniches I., Lemmen C., Cwik J.C., Kusch M., Labouvie H., Scholten N., Gerlach A., Stock S., Samel C., Hagemeier A. (2020). Evaluation of a complex integrated, cross-sectoral psycho-oncological care program (isPO): A mixed-methods study protocol. BMJ Open.

[22] Patton M.Q. (2002). Qualitative Research & Evaluation Methods.

[23] Mayring P. (2015). Qualitative Inhaltsanalyse: Grundlagen und Techniken.

[24] VERBI Software (2020). MAXQDA 2020, Computer Program.

[25] Luig T., Asselin J., Sharma A.M., Campbell-Scherer D.L. (2018). Understanding Implementation of Complex Interventions in Primary Care Teams. J. Am. Board Fam. Med..

[26] Krieger T., Boumans N., Feron F., Dorant E. (2020). The development of implementation management instruments for a new complex stroke caregiver intervention based on systematic stakeholder and risk analyses. Scand. J. Caring Sci..

[27] Greenhalgh T., Robert G., Macfarlane F., Bate P., Kyriakidou O. (2004). Diffusion of innovations in service organizations: Systematic review and recommendations. Milbank Q..

[28] Eboreime E.A., Eyles J., Nxumalo N., Eboreime O.L., Ramaswamy R. (2019). Implementation process and quality of a primary health care system improvement initiative in a decentralized context: A retrospective appraisal using the quality implementation framework. Int. J. Health Plan. Manag..

[29] Scheffler R.M., Arnold D.R. (2019). Projecting shortages and surpluses of doctors and nurses in the OECD: What looms ahead. Health Econ. Policy Law.

[30] WHO (2016). Global Strategy on Human Resources for Health: Workforce 2030.

[31] Fitzgerald L.A., van Eijnatten F.M. (2002). Reflections: Chaos in organizational change. J. OrgChange Mgmt.

[32] Plsek P.E., Wilson T. (2001). Complexity, leadership, and management in healthcare organisations. BMJ.

[33] Edmondson A.C., Bohmer R.M., Pisano G.P. (2001). Disrupted Routines: Team Learning and New Technology Implementation in Hospitals. Adm. Sci. Q..

[34] Safran D.G., Miller W., Beckman H. (2006). Organizational dimensions of relationship-centered care. Theory, evidence, and practice. J. Gen. Intern. Med..

[35] Bleijenberg N., de Man-van Ginkel J.M., Trappenburg J.C.A., Ettema R.G.A., Sino C.G., Heim N., Hafsteindóttir T.B., Richards D.A., Schuurmans M.J. (2018). Increasing value and reducing waste by optimizing the development of complex interventions: Enriching the development phase of the Medical Research Council (MRC) Framework. Int. J. Nurs. Stud..

[36] Abraham C., Denford S., Smith J., Dean S., Greaves C., Lloyd J., Tarrant M., White M., Wyatt K., Richards D.A., Rahm Hallberg I. (2015). Designing Interventions to Change Health-related Behaviour. Complex interventions in health: An overview of methods.

[37] Campbell N.C., Murray E., Darbyshire J., Emery J., Farmer A., Griffiths F., Guthrie B., Lester H., Wilson P., Kinmonth A.L. (2007). Designing and evaluating complex interventions to improve health care. BMJ.

[38] Caron F. (2014). Project planning and control: Early engagement of project stakeholders. J. Mod. Proj. Manag..

[39] Rychetnik L., Frommer M., Hawe P., Shiell A. (2002). Criteria for evaluating evidence on public health interventions. J. Epidemiol. Community Health.

[40] Young L. (2006). Participatory action research (PAR): A research strategy for nursing?. West. J. Nurs. Res..

[41] Krieger T., Floren M., Feron F., Dorant E. (2021). Optimising a complex stroke caregiver support programme in practice: A participatory action research study. Educ. Action Res..

[42] Jenny G., Bauer G., Vinje H., Vogt K., Torp S., Mittelmark M.B., Sagy S., Eriksson M., Bauer G.F., Pelikan J.M., Lindström B., Espnes G.A. (2017). The Application of Salutogenesis to Work. The Handbook of Salutogenesis: The Salutogenic Model: The Role of Generalized Resistance Resources.

[43] Brauchli R., Jenny G.J., Füllemann D., Bauer G.F. (2015). Towards a Job Demands-Resources Health Model: Empirical Testing with Generalizable Indicators of Job Demands, Job Resources, and Comprehensive Health Outcomes. Biomed Res. Int..

[44] Schaufeli W.B., Bakker A.B. (2004). Job demands, job resources, and their relationship with burnout and engagement: A multi-sample study. J. Organiz. Behav..

[45] Antonovsky A., Kalimo R., El-Batawi M.A., Cooper C.L. (1987). Health promoting factors at work: The sense of coherence. Psychosocial Factors at Work and Their Relation to Health.

[46] Vinje H.F., Mittelmark M.B. (2006). Deflecting the path to burnout among community health nurses: How the effective practice of self-tuning renews job engagement. Int. J. Ment. Health Promot..

[47] Bakibinga P., Vinje H.F., Mittelmark M.B. (2012). Self-tuning for job engagement: Ugandan nurses’ self-care strategies in coping with work stress. Int. J. Ment. Health Promot..

[48] Vinje H.F., Ausland L.H. (2013). Salutogenic presence supports a health-promoting work life. Soc. Tidskr..

[49] Vinje H.F., Mittelmark M.B. (2008). Community nurses who thrive: The critical role of job engagement in the face of adversity. J. Nurses Prof. Dev..

[50] Stoyanova K., Stoyanov D.S. (2021). Sense of Coherence and Burnout in Healthcare Professionals in the COVID-19 Era. Front. Psychiatry.

